# Nrf2/ARE Pathway Modulation by Dietary Energy Regulation in Neurological Disorders

**DOI:** 10.3389/fphar.2019.00033

**Published:** 2019-02-04

**Authors:** Andrea Rodrigues Vasconcelos, Nilton Barreto dos Santos, Cristoforo Scavone, Carolina Demarchi Munhoz

**Affiliations:** ^1^Laboratory of Molecular Neuropharmacology, Department of Pharmacology, Institute of Biomedical Science, University of São Paulo, São Paulo, Brazil; ^2^Laboratory of Neuroendocrinopharmacology and Immunomodulation, Department of Pharmacology, Institute of Biomedical Science, University of São Paulo, São Paulo, Brazil

**Keywords:** Nrf2, dietary energy restriction, high-fat diet, aging, Alzheimer’s disease, Parkinson’s disease, multiple sclerosis, cerebral ischemia

## Abstract

Nuclear factor erythroid 2-related factor 2 (Nrf2) regulates the expression of an array of enzymes with important detoxifying and antioxidant functions. Current findings support the role of high levels of oxidative stress in the pathogenesis of neurological disorders. Given the central role played by Nrf2 in counteracting oxidative damage, a number of studies have targeted the modulation of this transcription factor in order to confer neuroprotection. Nrf2 activity is tightly regulated by oxidative stress and energy-based stimuli. Thus, many dietary interventions based on energy intake regulation, such as dietary energy restriction (DER) or high-fat diet (HFD), modulate Nrf2 with consequences for a variety of cellular processes that affect brain health. DER, by either restricting calorie intake or meal frequency, activates Nrf2 thereby triggering its protective effects, whilst HFD inhibit this pathway, thereby exacerbating oxidative stress. Consequently, DER protocols can be valuable strategies in the management of central nervous system (CNS) disorders. Herein, we review current knowledge of the role of Nrf2 signaling in neurological diseases, namely Alzheimer’s disease, Parkinson’s disease, multiple sclerosis and cerebral ischemia, as well as the potential of energy intake regulation in the management of Nrf2 signaling.

## Introduction

It is widely recognized that oxidative stress plays a key role in CNS physiology and pathophysiology (Patel, 2016). Free radicals are constantly produced and are required at physiological levels for signaling and plasticity in the healthy brain. Conversely, their accumulation due to impaired cellular antioxidant defenses or excessive production that exceeds the cell’s antioxidant capability can result in neurotoxicity and cell death, which if continued will ultimately lead to pathological processes (Cai et al., 2011). For this reason, oxidative stress has been extensively studied as a therapeutic target to treat brain diseases (Patel, 2016).

It is important to note that the brain can be highly susceptible to oxidative damage, due, in part, to its elevated oxygen demand, the presence of high amounts of polyunsaturated fatty acids that are easily targeted by free radicals, and lower levels of antioxidant enzymes compared to other organs (Ahmad et al., 2017; Mecocci et al., 2018). Some stable products of lipid peroxidation in the CNS are substantial oxidative stress biomarkers largely used in studies involving neurological and neurodegenerative disorders. These oxidation products include isoprostanes and neuroprostanes, derived from the non-enzymatic oxidation of arachidonic acid and decosahexanoic acid, respectively (Reed, 2011).

Aging leads to a gradual increase in brain oxidative stress, which is accompanied by reduced antioxidant defenses and lower levels of neurogenesis (Uttara et al., 2009). Aging is the main risk factor for neurodegenerative disorders (Niccoli and Partridge, 2012), which accounts for 12% of total deaths worldwide (World Health Organization [WHO], 2011; Chen et al., 2016b). Both acute and chronic inflammatory processes reciprocally interact with oxidative stress, with these factors being important to the etiology and course of a wide array of neurological conditions, such as AD, PD, MS, and stroke, as well as to the process of aging *per se* (Sandberg et al., 2014).

**FIGURE 1:**
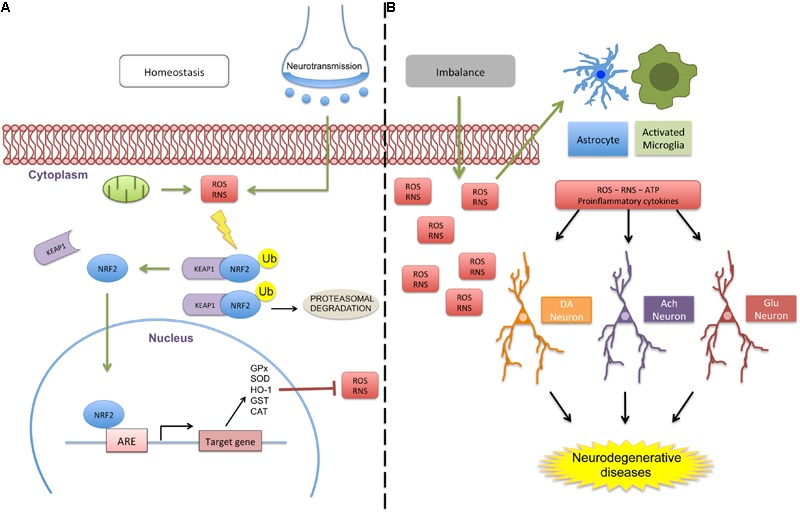
Schematic representation of Nrf2 signaling in homeostasis and a deregulated environment. **(A)** Oxidative molecules (e.g., ROS and RNS) produced by cellular respiration or neurotransmission activate the protective antioxidant pathway by dissociation of the Nrf2/KEAP1 complex. When dissociated from the cytosolic protein KEAP1, Nrf2 translocates to the cell nucleus, triggering the expression of several homeostatic genes with the ARE sequence in their promoters, including GPx, SOD, HO-1, GST, and CAT. When inactivated, Nrf2 is sequestered by KEAP1 and targeted for ubiquitination and proteasomal degradation. **(B)** Altered homeostasis promotes excessive ROS/RNS production that can activate glial cells (astrocytes and microglia) that release proinflammatory and danger molecules patterns, which disrupts neuronal communication and the nature of glial activities. Green arrows represent activation and truncated red lines, inhibition (abbreviations: ACh, acetylcoline; DA, dopamine; CAT, catalase; Glu, glutamate; GPx, Glutathione Peroxidase; GST, glutathione S-transferase; HO-1, heme oxigenase 1; RNS, reactive nitrogen species; ROS, reactive oxygen species; SOD, superoxide dismutase; Ub, ubiquitin; ATP, adenosine triphosphate).

Aging is also associated to a progressive reduction in Nrf2 activity (Cuadrado, 2016). Interestingly, long-lived animal species have higher Nrf2 signaling levels, highlighting the importance of Nrf2 protection against aging and aging-related diseases (Bruns et al., 2015). Nrf2 is pivotal in the regulation of cellular redox status, modulating the expression of more than 200 downstream genes encoding Phase II response enzymes during oxidative challenge, including HO-1, GST, CAT, SOD, and NQO1 (Nguyen et al., 2009; Sun et al., 2017). The phase II response in an evolutionary conserved adaption to a broad range of stressors and is intimately linked to the organism’s antioxidant defenses, detoxification, and cellular resilience (Hine and Mitchell, 2012). A broad array of published data show that the upregulation of Nrf2 target genes in the CNS can render neurons more resistant to excitotoxic and oxidative insults (Chen et al., 2000; Satoh et al., 2006; Giordano et al., 2007; Tanito et al., 2007; Lim et al., 2008). Nrf2 not only modulates antioxidant defense genes, but also genes that have autophagic and anti-inflammatory properties as well as glucose and lipid metabolism effects (Bruns et al., 2015; Tebay et al., 2015). Nrf2 activation leads to its translocation to the cell nucleus where it triggers the expression of target genes that contain the ARE DNA regulatory sequence in their promoter region (Jaiswal, 2004). The Nrf2/ARE pathway is modulated by the KEAP1. In basal conditions, this protein acts as a Nrf2 repressor, binding to Nrf2 and maintaining it in the cell cytoplasm (Satoh et al., 2006). This regulatory protein also directs Nrf2 to ubiquitination and degradation by proteasomes, thereby limiting its basal cellular levels (Sun et al., 2017) (Figure 1).

**FIGURE 2:**
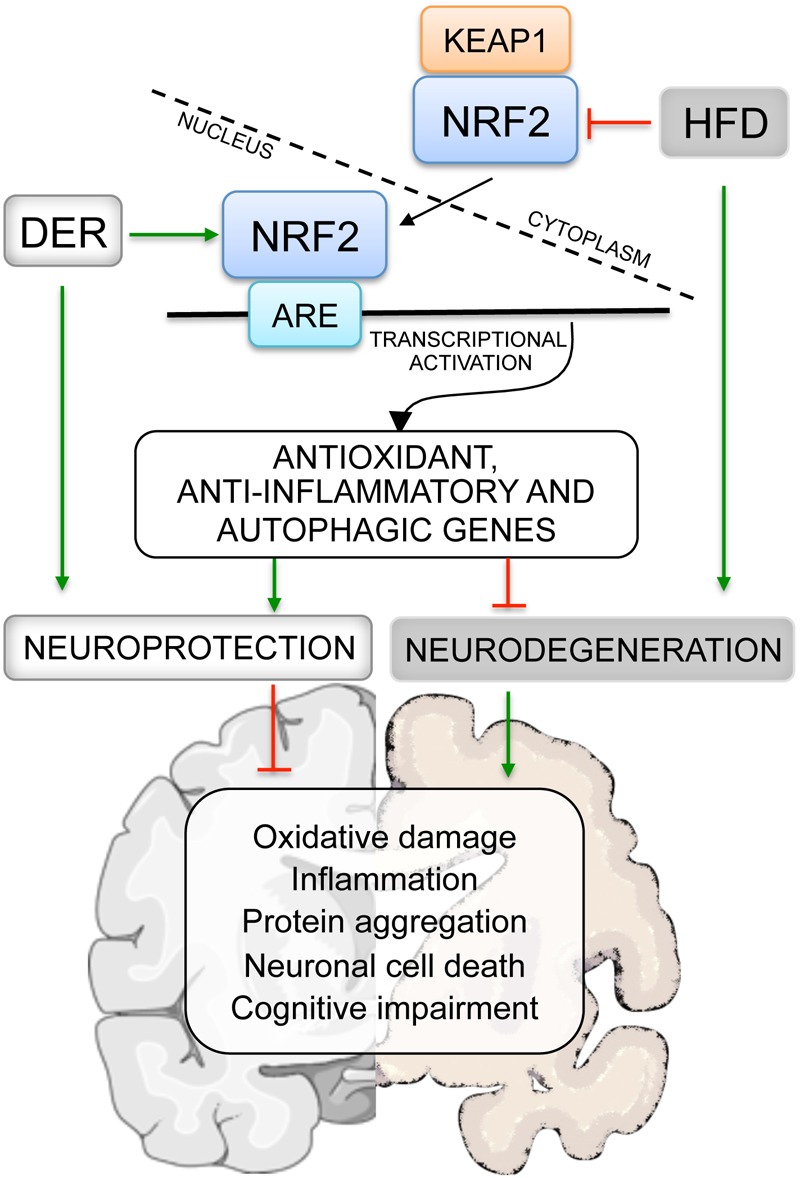
The role of Nrf2 modulation by dietary interventions on brain health. HFD inhibit Nrf2 in the brain, whereas DER is able to activate this transcription factor. When activated, Nrf2 triggers the expression of several neuroprotective genes that counteract oxidative stress and neuroinflammation, preventing the onset of neurological disorders and underlying pathological processes. Green arrows represent activation and truncated red lines, inhibition (abbreviations: DER, dietary energy restriction; HFD, high-fat diet).

Many dietary interventions modulate Nrf2. DER and high energy consumption are two of the most studied strategies for energy status regulation, and both act to modulate tNrf2 activity. DER increases Nrf2 activity, in contrast to high energy consumption. DER, induced by chronically or intermittently restricted calorie consumption, subjects neurons to an energetic stress that triggers the Nrf2/ARE pathway and thereby induces many beneficial effects on health and longevity, including the prevention of neurological diseases (Mattson, 2012). In contrast, a plethora of animal and human studies show that a HFD, and associated obesity, enhance inflammation and oxidative stress, resulting in a raised overall mortality and higher incidence of many neurological disorders (Dorrance et al., 2014; Michel, 2016; Mazon et al., 2017; Alfredsson and Olsson, 2018) (Figure 2). This chapter focuses on the Nrf2/ARE pathway regulation by dietary interventions and its protective role in the CNS against metabolic, excitotoxic, and oxidative insults, with relevance to AD, PD, MS, and cerebral ischemia.

## Nrf2/ARE Pathway and Neurological Diseases

Both *in vitro* and *in vivo* neurodegenerative models have demonstrated that Nrf2 activation promotes neuroprotective effects (Calkins et al., 2009). However, as noted above, Nrf2 activity declines with age, consequently decreasing the protection afforded by antioxidant enzymes activity. Such Nrf2/ARE signaling impairment renders the organism and the brain more susceptible to oxidative injury, abnormal protein aggregation and neurodegeneration (Itoh et al., 1997; Suh et al., 2004; Shih and Yen, 2007; Morrison et al., 2010; Safdar et al., 2010; Cuadrado, 2016). A number of studies have underscored the importance of the Nrf2/ARE pathway to the pathogenesis and treatment of neurological disorders, including PD, AD, MS, and cerebral ischemia, as described below.

### Alzheimer’s Disease

Alzheimer’s disease is the leading cause of senile dementia, characterized by progressive cognitive impairment and memory loss. In the United States, it is estimated that 5.7 million people have AD in 2018 and this number is predicted to reach 14 million by 2050, due to rises in longevity (Alzheimer’s Association, 2018). Classically, AD has been thought to be driven by the accumulation of amyloid-β peptide (Aβ) aggregates and neurofibrillary tangles composed of hyperphosphorylated tau proteins (Arriagada et al., 1992; Selkoe, 1994). However, there is a growing appreciation of a role for other processes in AD etiology and course, including changes in oxidative stress (Mecocci et al., 2018). Oxidative stress markers have long been shown to be increased in AD. Patients with mild cognitive impairment, often a forerunner of AD, also show raised levels of oxidative damage and reduced antioxidant defenses, in comparison to healthy controls (Migliore et al., 2005; Bermejo et al., 2008; Mangialasche et al., 2009; Padurariu et al., 2010). Markers of oxidative stress are all increased in AD, including 4-hydroxynonenal (a product of lipid peroxidation) (Bradley et al., 2012; Di Domenico et al., 2017), and protein nitration and carbonylation (Perluigi et al., 2009;Aluise et al., 2011). These changes are often accompanied by a decline in antioxidant defenses, which contributes to such heightened oxidative stress in AD patients (Kim et al., 2006).

Nrf2 levels are decreased in the AD brain (Kanninen et al., 2008). This is supported by data from preclinical models of AD, which indicate reduced brain Nrf2 expression levels (Carvalho et al., 2015; Liu et al., 2016; Manczak et al., 2018), with Nrf2 activation mitigating neuronal apoptosis and spatial memory impairment (Dong F. et al., 2017). Several *in vivo* and *in vitro* studies also show that the activation of Nrf2/HO-1 signaling cascade by flavonoids and microRNA-302 affords protection against neuronal toxicity induced by Aβ (Zou et al., 2013; Kwon et al., 2015; Li H.H. et al., 2016; Wang et al., 2016). Moreover, Nrf2 knockout mice have an impaired clearance of phosphorylated tau by autophagy, contributing to heightened tau aggregation and accumulation in the brain, a well-known hallmark of AD (Jo et al., 2014). In an AD murine model, Nrf2 deletion also results in impaired autophagy, and therefore a decrease in the ability of a cell to clear debris (Joshi et al., 2015). These findings highlight the importance of Nrf2 in AD pathophysiology as well as indicating its potential therapeutic utility in AD.

In contrast, Raina et al. (1999) reported increased levels of NQO1 in AD human brains, suggesting Nrf2 activation. Similarly, three common Nrf2 target genes (p62, HO-1, and GCLM) are upregulated in AD brains (Tanji et al., 2013). Such contrasting results may be due to differential effects on Nrf2 levels at different AD stages, perhaps indicative of an upregulation of antioxidant defenses to counteract oxidative stress in early AD stages, whilst the loss of endogenous antioxidants and Nrf2/ARE pathway activity may be more evident in latter phases of the disease (Sun et al., 2017).

### Parkinson’s Disease

After AD, PD is the second most prevalent neurodegenerative disease. PD is typically characterized by progressive movement disorders (rigidity, resting tremor, postural instability, hypokinesia, and bradykinesia) as well as by variable degrees of cognitive dysfunction and dementia (Goris et al., 2007; Tufekci et al., 2011). A number of different brain regions show atrophy in PD, with classic PD symptomatology mediated by the loss of dopamine neurons in the SN. An accumulation of α-synuclein protein is common, in association with proteasome and mitochondria impairment (Mattson, 2012).

As with AD, the etiology of PD still awaits clarification, with a number of studies indicating that oxidative stress is an important contributor to PD pathogenesis and course, including via increased membrane lipid peroxidation and protein damage (Duan and Mattson, 1999; Jenner, 2007; Cuadrado et al., 2009). Sources of oxidative stress in PD brain include chronic neuroinflammation, the metabolism of dopamine producing cytotoxic ROS, and mitochondrial impairment (Blesa et al., 2015). Nrf2 is an emerging target to counteract PD-related neuronal cell death, given its regulation of a plethora of cytoprotective genes with anti-inflammatory and antioxidant properties, as well as its role in optimizing mitochondria biogenesis (Tufekci et al., 2011). Data from human post-mortem PD brains and Nrf2 knockout mice indicate an association between Nrf2/ARE pathway dysfunction and PD pathogenesis (Tufekci et al., 2011). Such data shows that Nrf2 deficiency increases the sensitivity of mice to PD neurotoxins (Jakel et al., 2007; Chen et al., 2009; Colton, 2009), whereas the induction of this transcription factor in astrocytes exerts a protective effect against brain damage in the 6-OHDA model of PD (Jakel et al., 2007). In post-mortem brains of PD patients, the proteins p62 and NQO1 were partially sequestered in Lewy bodies, indicating a compromised neuroprotective capacity of Nrf2 (Lastres-Becker et al., 2016). In the same study, Nrf2 pharmacological activation by dimethyl fumarate in a murine PD model protected SN neurons against α-synuclein toxicity, an effect not evident in Nrf2-knockout mice (Lastres-Becker et al., 2016). Jazwa et al. (2011) also showed that Nrf2 activation upregulates brain HO-1 and NQO1 expression and prevents SN neuronal death as induced by MPTP, a neurotoxin PD model. Furthermore, the link between Nrf2 and PD is also supported by studies showing that antiparkinsonian drugs (e.g., apomorphine, deprenyl, and bromocriptine) can activate the Nrf2/ARE pathway and Nrf2-dependent gene expression, preventing cell oxidative damage and neurodegeneration (Hara et al., 2006; Nakaso et al., 2006; Lim et al., 2008; Kabel et al., 2018).

In human PD brain tissue, Ramsey et al. (2007) showed that hippocampal neurons and glia from CA1 region had lower nuclear Nrf2 when compared to age-matched healthy controls. In contrast, SN neurons of PD patients had higher Nrf2 nuclear localization, indicative of Nrf2 activation, although not sufficient to counteract neurodegeneration in these individuals (Ramsey et al., 2007). Similarly, Nrf2-dependent gene expression is decreased in the striatum but increased in the SN of the MPTP model (Ramsey et al., 2007). A recent study also reported increased Nrf2 activation in SN of mice treated with MPTP, prior to the onset of neurodegeneration (Rizzi et al., 2018).

Further research is required to clarify why the increased Nrf2-dependent gene expression and nuclear translocation in the SN reported in the aforementioned studies is incapable of protecting neurons against oxidative damage. A number of studies indicate a crucial role for Nrf2 activation in glial cells (Jakel et al., 2007; Chen et al., 2009), suggesting that Nrf2 may need to be activated in glia in order to exert its protective effects in PD and PD models. Ramsey study did not report glial Nrf2 nuclear translocation in the SN of PD brains (Ramsey et al., 2007) and *in vitro* studies indicate that neuronal Nrf2 activation, even in the absence of glia, induces neuroprotection against oxidative damage triggered by parkinsonism-inducing neurotoxins (Lee et al., 2003; Cao et al., 2005; Hara et al., 2006; Jakel et al., 2007; Wruck et al., 2007; Hwang and Jeong, 2008; MacKenzie et al., 2008; Satoh et al., 2009; Niso-Santano et al., 2010). Overall, such work indicates that increased Nrf2 activity in both glia and neurons is important to neuronal survival in PD (Tufekci et al., 2011).

### Cerebral Ischemia

Cerebrovascular diseases are the second leading cause of death worldwide. Without intervention, the cases of deaths worldwide are estimated to increase from 6.5 million in 2015 to 7–8 million in 2030 (Strong et al., 2007). Ischemic stroke is characterized by decreased blood flow in the brain, causing injury to brain tissues and impairing normal neurological functioning (Jauch et al., 2013; Ding et al., 2017).

The reduced delivery of nutrients and oxygen after stroke decreases tissue pH, resulting in impairment of the mitochondrial electron transport chain activity and subsequent diminished ATP production. Subsequently, a cascade of events follows that culminates in raised intracellular Na^+^, Ca^2+^, Cl^-^ concentrations, and K^+^ efflux. The neuronal cations increase leads to excessive depolarization and the release of excitatory neurotransmitters, such as glutamate, causing excitotoxicity and triggering oxidative and cell death pathways (Nicholls, 2009).

Increased oxidative stress is evident in stroke. Murine models, subjected to ischemia-reperfusion, show a high concentration of superoxide and peroxynitrite, as well as metalloproteinase-9 production and BBB breakdown (Gursoy-Ozdemir et al., 2004). The anti-apoptotic and anti-oxidative effects of the Nrf2 signaling pathway in stroke have been noted. Nrf2 activators are able to reduce oxidative stress and exert protective roles in models of stroke (Alfieri et al., 2011; Yang et al., 2018). Astrocyte Nrf2 activation is important to the release of antioxidants, such as glutathione, which protect neurons from free radicals produced during ischemia (Alfieri et al., 2011).

Recent work shows Nrf2 to induce angiogenesis via HO-1 (Ding et al., 2014) and VEGF signaling (Huang Y. et al., 2018), which is supported by the work of Bai and coworkers who showed that epigallocatechin-3-gallate, the major effective polyphenol in green tea, promotes angiogenesis and decreases oxidative stress via MAPK/Nrf2/VEGF activation, following tMCAO (Bai et al., 2017; Li L. et al., 2016).

In addition to antioxidant effects, Nrf2/ARE activation can afford protection against the neuroinflammation evident following ischemia. In the ischemic brain, ROS, as with DAMPS, can activate the NLRP3 inflammasome, thereby converting pro-interleukin (IL)-18 and pro-IL-1β into mature IL-18 and IL-1β forms, which are then released (Inoue et al., 2012). Glial cells are important drivers of neuroinflammation and NLRP3 activation (Frank et al., 2007; Gavillet et al., 2008). Xu et al. (2018) showed that the Nrf2/ARE pathway protects against the oxidative stress-induced NLRP3 activation in the BV2 microglial cell line exposed to OGDR. Moreover, neonatal rats treated with LPS 24 h prior to hypoxia-ischemia, were protected against neuropathological effects, whereas LPS administration 72 h before hypoxia-ischemia increased brain damage, which was prevented by treatment with *N*-acetylcysteine, a glutathione precursor (Wang et al., 2007). Such data support the idea that Nrf2 upregulation varies according to the time of ischemia exposure, as well as cell type (glial cells or neurons) and anti-oxidant reserve. Such data suggests complex effects, although it is clear that Nrf2-related pathways are important molecular targets for pharmacologic intervention in the management of ischemic stroke.

### Multiple Sclerosis

Multiple sclerosis is the most frequent chronic neuroinflammatory disease of the CNS, characterized by demyelination, as well as focal inflammatory lesions in the brain and spinal cord, which culminates in axonal damage (Lassmann, 2011b). MS is also associated with gray matter synaptic loss and decreased neurogenesis, which contributes to cognitive impairment. MS clinical symptoms include sensory or motor impairment, fatigue, ataxia, and spasticity, as well as cognitive impairment (Chiaravalloti and DeLuca, 2008; Lublin et al., 2014). EAE is the most common experimental model of MS (Simmons et al., 2013). Activated macrophages and microglial cells can produce an array of neurotoxic factors, including proteases, nitric oxide, and ROS, with this being evident in both MS and the EAE model (Glass et al., 2010). Generation of ROS and other free radicals by macrophages is involved in demyelination and axonal damage in EAE. Evidence of lipid peroxidation, as a consequence of oxidative stress, can be detected in the exhaled breath samples of MS patients. Additional studies have shown decreased levels of antioxidant enzymes in blood and cerebrospinal fluid of MS patients (Lassmann, 2011a,b, 2014; Witte et al., 2014). Such data highlights the importance of heightened levels of immune-inflammatory activity and oxidative stress in MS.

Therapeutic strategies in the treatment of MS have primarily focused on dampening the heightened immune-inflammatory activity that is present, including by the use of: imunossupressors, such as synthetic glucocorticoids (e.g., dexamethasone, prednisone, and methylprednisone); monoclonal antibodies (Natalizumab); inhibitors of cell egress from lymphoid organs (Fingolimod), modulators of pro- and anti-inflammatory cytokines (interferon-beta); and microglial inhibitors (acetate glatiramer). However, all of these treatments have shown limited efficacy. Antioxidant therapies have proved to have some utility when used as adjuvants in MS treatment, with endogenous Nrf2 being an important treatment target for the induction of endogenous antioxidants (Kappos et al., 2010; Lim et al., 2014).

Classical treatment in MS has emphasized the role of heightened immune-inflammatory processes. As noted above, such processes are intimately linked to alterations in redox regulation. There is now a growing appreciation of the role the oxidant/antioxidant balance in MS. A growing body of data indicates an important role for ROS in MS, including: a central role in MS lesion development and progression and an initial contribution to the BBB breakdown and leukocyte infiltration that is crucial in early phases of lesion formation in MS (Van der Goes et al., 2001; Schreibelt et al., 2006). ROS can cause oxidative injury in CNS cells, including during demyelination, oligodendrocyte injury, and axonal degeneration. OPCs are highly vulnerable to oxidative injury due to their low levels of endogenous antioxidant enzymes and the relatively high levels of polyunsaturated fatty acids in the myelin sheets, which are more susceptible to lipid peroxidation (Juurlink et al., 1998; French et al., 2009). Furthermore, free radicals are able to impair the maturation of OPCs into myelin-forming mature cells by modulating the genes driving maturation and differentiation.

Antioxidant molecules and oxidative stress are upregulated in active brain lesions in MS patients, but not to a sufficient level to counteract the oxidative stress evident in MS brain tissue. NADPH oxidase subunits are strongly upregulated in the macrophages and microglia in active MS lesions, suggesting an important role for heightened ROS, including as mediated by an oxidative burst, in the pathophysiology of active lesions. Contributing to such damage are increased expression of inducible nitric oxide synthase (Bo et al., 1994; De Groot et al., 1997; Liu et al., 2001) and myeloperoxidase (Marik et al., 2007; Chen et al., 2008; Gray et al., 2008a,b; Wang et al., 2016) that are evident in the brains of MS patients.

Although a number of different cell types, such as macrophages, can have an antigen presenting capacity, DCs are the body’s major antigen presenting cells. There is growing body of data indicating that Nrf2 may regulate the differentiation and function of macrophages and DCs, and therefore in antigen presentation and the regulation of adaptive immune response. In fact, Nrf2 deficiency modifies the phenotype and function of DCs by increasing the expression of co-stimulatory molecules and consequently the antigen-specific T cell reactivity (Al-Huseini et al., 2013). Some of these Nrf2 effects may be mediated via alterations in mitochondrial functioning, as indicated above, including in the mitochondrial regulation of immune cell responses.

Nrf2 is strongly upregulated in active MS lesions, with the expression of Nrf2-responsive genes being predominantly found in areas of initial myelin destruction (Licht-Mayer et al., 2015). Analyses of MS inflammatory lesions showed upregulation of Nrf2 and its downstream antioxidant enzymes, HO-1 and NQO1. In support of the importance of such Nrf2-mediated changes, experimental studies in Nrf2-deficient EAE mice show an increase in disease signals and peripheral cell infiltration (Lim et al., 2014). Accordingly, Johnson et al. (2010) showed that the absence of Nrf2 exacerbates the development of EAE. Part of the effects associated with Nrf2 deficiency may be related to the reduced levels of HO-1. Indeed, mice with a myeloid-specific HO-1 deficiency exhibit a higher incidence of lesions, accompanied by activation of antigen-presenting cells and the infiltration of the pro-inflammatory t helper (Th)17 cells and myelin-specific T cells (Tzima et al., 2009). Knockdown of KEAP1 (Kobayashi et al., 2016) or treatment with a wide range of small molecules that activate Nrf2 (Buendia et al., 2016) inhibits the development and severity of EAE.

## Role of Nrf2/ARE Pathway Modulation by Dietary Interventions in Neurological Diseases

A growing literature shows Nrf2 to regulate the expression of genes that reduce gluconeogenesis and lipogenesis, whilst also increasing fatty acids β-oxidation and mitochondria activity. Such data indicates that Nrf2 activity intimately interacts with cellular nutritional and energetic status (Holmstrom et al., 2013; Ludtmann et al., 2014; Tebay et al., 2015). The multiple molecular pathways that may underpin such interactions have been reviewed previously (Tebay et al., 2015). The present article reviews the current knowledge of dietary interventions, namely DER and HDF, on Nrf2 and the relevance of this to neurological disorders.

### Dietary Energy Restriction

Dietary energy restriction, the decrease of food intake without malnutrition, is the most powerful intervention known to delay aging processes and extend lifespan (Pearson et al., 2008; Hine and Mitchell, 2012). Several studies have comprehensively highlighted the beneficial effects of DER on cognitive function, metabolic health, and longevity, which have been especially associated with the two main DER protocols: caloric restriction (CR) and IF (Horne et al., 2015; Vasconcelos et al., 2018). In CR protocol, calorie consumption is chronically decreased in 20–40% of the *ad libitum* intake, whilst IF involves a restriction in the frequency of food intake, giving periods of free food intake coupled to fasting periods. These protocols were shown to extend life- and health-span and to counteract numerous age-related diseases. The vulnerability of the CNS to age progression is frequently expressed in neurological disorders, such as AD, PD, and stroke (Martin et al., 2006; Logsdon et al., 2017).

Dietary energy restriction, as a mild stress of energy restriction to the organism, is considered a hormetic stimulus, which is defined as a low dose stressor that induces adaptive responses able to improve resistance to more severe stressors and diseases. Within this context, the energetic challenge to the brain induced by DER triggers beneficial outcomes, including neurogenesis, synapses strengthening, and new synapse formation (Calabrese et al., 2010; Hine and Mitchell, 2012; Horne et al., 2015). Molecularly, DER induces the activation of protective transcription factors, such as Nrf2, that activate the expression of Phase II detoxifying enzymes, thereby increasing neuronal resistance to oxidative stress and death, and hence lowering the risk of neurodegenerative disorders. DER, especially IF, upregulates genes encoding the antioxidant enzymes that are modulated by Nrf2, including GPx, SOD2, and HO-1 (Hine and Mitchell, 2012; Mattson, 2012).

Some of the benefits of CR protocol are associated with Nrf2/ARE pathway activation (Pearson et al., 2008; Martin-Montalvo et al., 2011; Bruns et al., 2015). One of the proposed mechanisms of DER utility is via a transient ROS increase to hormetic levels that may activate Nrf2 (Hine and Mitchell, 2012). For instance, fasting, and its consequential effects on insulin levels, results in a small, transient increase in oxidative stress, triggering activation of the Nrf2/ARE pathway and the upregulation of its target genes (Kim and Novak, 2007).

Various age-related diseases, including AD and PD, are linked to decreased Nrf2 activity and display symptom improvement after Nrf2 activation by DER (Hine and Mitchell, 2012). CR can counteract the age-related loss of cellular antioxidant defenses, partly by promoting the up-regulation of Nrf2/ARE-driven genes, including GST and NQO1, in a variety of body tissues and organs, including the liver and brain (Chen et al., 1994; Hyun et al., 2006). Furthermore, a recent study showed that 30% CR for 12 weeks can prevent neurotoxicity, oxidative damage, and cognitive impairment induced by acrolein. Acrolein has been proposed to be involved in AD etiology, with the efficacy of CR mediated, at least partly, through the amelioration of acrolein-induced depletion of hippocampal SOD levels, indicating a positive effect of this protocol on Nrf2 signaling (Huang Y.J. et al., 2018).

The age-induced Nrf2 dysfunction in BBB endothelial cells is also prevented by CR, shedding light in the cerebrovascular protective effects of this DER protocol (Csiszar et al., 2014). Importantly, these age-dependent endothelial alterations are thought to play a role in both vascular cognitive impairment and AD (Gorelick et al., 2011; Zlokovic, 2011; Lin et al., 2013). Accordingly, numerous substances proposed as “DER mimetics” (i.e., compounds shown to promote the beneficial effects of DER in the absence of food intake restriction), such as curcumin, resveratrol, and quercetin (Ingram et al., 2006), have been shown to increase longevity and slow down the aging process, at least in part via Nrf2 activation (Balogun et al., 2003; Chen et al., 2005; Hsieh et al., 2006; Tanigawa et al., 2007; Calabrese et al., 2010).

Several studies in murine and primate models of PD have demonstrated that DER can protect dopaminergic neurons, decrease motor dysfunction, and alleviate PD symptoms (Duan and Mattson, 1999; Maswood et al., 2004; Qiu et al., 2012; Griffioen et al., 2013), even when the DER protocol is initiated after the induction of PD by MPTP (Holmer et al., 2005). Published data also indicate that energy consumption profoundly impacts the progression of AD (Mattson, 2012), with DER decreasing brain Aβ accumulation in the APP transgenic murine model of AD (Patel et al., 2005; Wang et al., 2005) and aged primates (Qin et al., 2006). Furthermore, in a triple transgenic AD mice, both IF and CR protocols, when starting at 3 months of age (before symptoms onset), can counteract age-related cognitive impairment (Halagappa et al., 2007). Interestingly, in this study, CR, but not IF, reduced Aβ deposition in the brain. The authors suggest that the IF mechanism of action may involve the prevention of Aβ-mediated negative effects on cognitive function.

Glutathione is an important reducing agent of the phase II antioxidant response. Many genes involved in glutathione metabolism are modulated by Nrf2, including glutathione synthesis genes (GCLM, GCLC), GST, GPx, and GR. Several studies have reported an age-related disruption of the glutathione antioxidant system in rodents and humans, which may result in increased susceptibility to PD and AD (Rao et al., 1990; Samiec et al., 1998; Cho et al., 2003; Suh et al., 2004; Kennedy et al., 2005; Ballatori et al., 2009; Hine and Mitchell, 2012). Cho et al. (2003) showed that CR in rodents is able to prevent the age-related decrease in the levels of glutathione and glutathione-related enzyme activities. Furthermore, many studies showed that GST and GPx levels and activities can be augmented by both fasting and CR (Leakey et al., 1989; Cho et al., 2003; Pearson et al., 2008; Mitchell et al., 2010; Vazquez-Medina et al., 2011). In one of these studies, these effects were evident in mice subjected to 30% CR for 2–4 weeks or short periods of fasting, following ischemia reperfusion injury (Mitchell et al., 2010). Results from the CALERIE Trial of Human Caloric Restriction also showed that GPx activity is increased by 10–30% CR over 6 months in overweight individuals (Meydani et al., 2011).

NQO1 is another important endogenous antioxidant defense enzyme modulated by Nrf2. Altered expression of NQO1 is correlated with many pathologies, including AD and PD (Lastres-Becker, 2017; Chhetri et al., 2018). Long term DR can increase NQO1 expression, resulting in enhanced antioxidant defenses in the brain and liver of aged rats (De Cabo et al., 2004; Hyun et al., 2006). This effect was also observed in studies using DER mimetics (Zhu et al., 2005; Higgins et al., 2009; Son et al., 2010).

DER and its mimetics can also counteract damage following ischemia reperfusion injury (Go et al., 1988; Khan et al., 2006; Saleh et al., 2010; Peng et al., 2012). After experimental stroke in rodents, 70 days of DER leads to a substantial attenuation of cognitive dysfunction as well as increasing hippocampal cell survival (Roberge et al., 2008). These effects seem, in part, to involve the Nrf2-triggering effect of DER (Mattson, 2012). Fasting for up to 4 days or 30% CR for 2–4 weeks results in augmented HO-1 expression and attenuates ischemic damage of the brain, liver, and kidney in rodents (Go et al., 1988; Mitchell et al., 2010; Verweij et al., 2011). Moreover, *in vivo* or *in vitro* data shows that DER mimetics, such as curcumin and plumbagin, also increase HO-1 expression and render rodents more resistant to acute stressors and oxidative damage (Farombi et al., 2008; Son et al., 2010). In one of these studies, plumbagin pre-treatment in a murine model of focal ischemic stroke led to the attenuation of brain injury and neurological deficits. These effects were attributed to Nrf2/ARE activation, given that Nrf2 knockdown prevents such neuroprotective effects (Son et al., 2010).

Current MS treatments have poor efficacy, both for symptom relief and disease progression (Lublin et al., 2014). Genetic risk factors do not fully explain the development of MS, with a number of environmental factors, including infections, smoking, low vitamin D levels and obesity, associated with increased incidence of MS (Ascherio, 2013). A number of studies show childhood/young adulthood obesity to be a risk factor for MS (Munger et al., 2009; Hedstrom et al., 2012, 2014; Langer-Gould et al., 2013). The chronic inflammatory state that is evident in obesity can promote autoimmunity though adipokine production (Calder et al., 2011). The effects of diet on the gut microbiome is thought to contribute to this, via the regulation of pro- and anti-inflammatory responses that regulate DC activation, MHC II presentation, and T cell differentiation in the gut (Goto et al., 2014; Furusawa et al., 2015). The gut microbiome in both RRMS patients and EAE models is altered compared to healthy controls (Chen et al., 2016a; Jangi et al., 2016). Several dietary habits such as high salt intake or long chain fatty acid intake, have been recently recognized as environmental contributors to the pathogenesis of MS and EAE, by expanding TH1 and TH17 cells (Berer et al., 2011; Kleinewietfeld et al., 2013).

Chronic CR has a potent anti-inflammatory effect, protecting against EAE symptoms (Piccio et al., 2008; Meydani et al., 2016). In these studies, CR reduced inflammation, demyelination, and neurodegeneration ameliorating relapsing-remitting EAE in SJL mice and progressive EAE in C57BL/6 mice. CR lowers plasma IL-6 concentration in the course of EAE accompanied by a decrease in leptin, suggesting CR-mediated alterations in the gut-brain axis and associated changes in gut-mediated inflammatory processes (Piccio et al., 2008). As leptin induces Th1 cell differentiation, such changes are likely to lower the levels of proinflammatory cytokine production (Matarese et al., 2005). Accordingly, MS patients showed an increased concentration of leptin in the serum and cerebrospinal fluid, associated with reduced levels of CD4 (+) CD25+ regulatory T cells and augmented INF-γ release (Matarese et al., 2005).

Recent studies indicate that MS show characteristics of metabolic disease, with SPMS patients showing elevated peroxisomal metabolites (PlsEtn) and increased mitochondrial stress metabolites (VLCFA-PtdEtn), when compared to control patients (Senanayake et al., 2015). Furthermore, SPMS patients showed reduced seric anti-inflammatory hydroxylated long-chain fatty acids called gastro-intestinal tract acids (GTAs), suggesting a diminished protection against MS-related inflammation. In addition, the oxidative stress-induced mitochondrial dysfunction in MS may provide biomarkers for the susceptibility to, and progression of, MS.

Importantly, although Nrf2/ARE signaling plays a critical role in cellular detoxification responses and prevention of age-related diseases, excessive Nrf2 activation has deleterious consequences. This is supported by studies showing KEAP1 knockout to be lethal in mice as a consequence of excessive constitutive Nrf2 activation (Wakabayashi et al., 2003), with Nrf2 overexpression (DeNicola et al., 2011; Lister et al., 2011) or mutation of KEAP2 (Zhang et al., 2010) promoting tumorigenesis. Consequently, pharmacological activation of Nrf2 may be dangerous, with the physiological activation of Nrf2 by DER likely to be a safer alternative.

### High Energy Consumption

The Western lifestyle are frequently more sedate with greater levels of over-eating, characterizing a condition of chronic positive energy balance (Martin et al., 2009; Mattson, 2012). In contrast to DER, high-energy consumption is associated with many negative impacts on overall health and longevity, resulting in increased morbidity and mortality (Maffei et al., 1995; Caro et al., 1996; Herrmann et al., 2001). Consequently, being overweight or obesity has reached epidemic proportions, being now classed as the fifth largest cause of death worldwide (Razay et al., 2006; Procaccini et al., 2016). In fact, the World Health Organization estimated that the global burden of obesity and overweight was over 300 million and 1 billion of adults, respectively (World Health Organization [WHO], 2011). Obesity, in turn, is an important risk factor for diabetes, a metabolic disease associated to chronic hyperglycemia and an array of other complications (Tebay et al., 2015). A HFD (typically 40–60% of total calorie intake from fat) in humans and animals results in an augmented vulnerability to an array of medical conditions, including many psychiatric disorders where risk positively correlates with BMI (Lopes et al., 2001; Degirmenci et al., 2015). High BMI in human subjects is also linked to reduced blood flow in brain regions important for cognitive function (Willeumier et al., 2011) and reduced brain integrity (Gazdzinski et al., 2008; Stanek et al., 2011). Even a short-term HFD, for only 7 days, can cause cognitive impairment in humans (Edwards et al., 2011).

**FIGURE 3:**
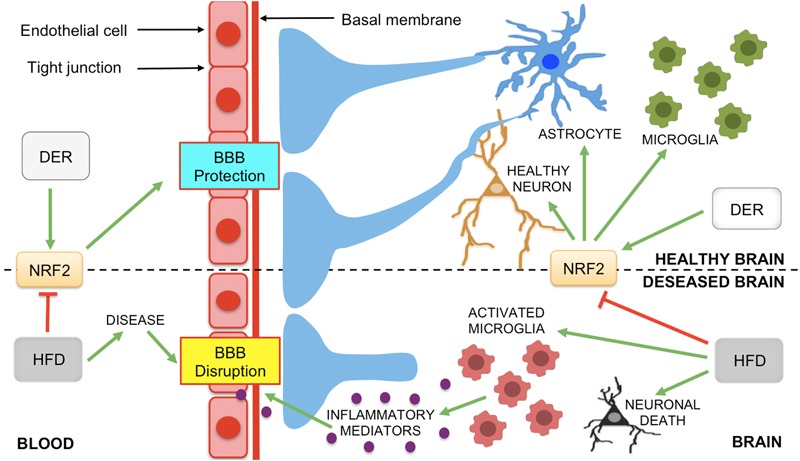
The role of NRF2 and dietary interventions (DER and HFD) on the modification of the BBB. Neurological diseases, such as AD, PD, MS, and ischemic stroke, can lead to BBB disruption. However, Nrf2 protects neurons and the BBB against oxidative stress and inflammation-induced damage. Astrocytes, microglia and neurons produce Nrf2 that activates the expression of antioxidant and anti-inflammatory genes to the maintenance of neuronal health and BBB integrity. Nrf2 protective effects on BBB can be modulated by DER and HFD, modifying the release of inflammatory mediators by glial cells in neurodegenerative diseases. Green arrows represent activation and truncated red lines, inhibition (abbreviations: BBB, blood-brain barrier; DER, dietary energy restriction; HFD, high-fat diet, MS, multiple sclerosis; PD, Parkinson’s disease; DER, dietary energy restriction; HFD, high-fat diet).

Accumulating data clearly shows that obesity is a risk factor for cognitive decline, dementia and neurodegenerative diseases, such as AD and PD (Mazon et al., 2017). One of the mechanisms by which diet-induced obesity can lead to neurological disorders is through increased neuroinflammation and ROS production and the downregulation of endogenous antioxidant enzymes, resulting in increased oxidative damage to the CNS (Edwards et al., 2011; Matsuda and Shimomura, 2013; Guillemot-Legris and Muccioli, 2017; Mazon et al., 2017). Diet-induced obesity can also lead to neurological disorders through microglia activation and BBB disruption, which can trigger neuroinflammation and synaptic impairment, thereby resulting in cognitive decline and neurodegeneration (Pistell et al., 2010; Zlokovic, 2011; Knight et al., 2014; Tucsek et al., 2014) (Figure 3).

Nrf2 activation can improve energy expenditure and prevent weight gain in HFD mice (Shin et al., 2009). Moreover, Nrf2/HO-1 pathway activation can ameliorate long-term HFD-induced cognitive impairment, inflammation, as well as the accumulation of Aβ and hyperphosphorilated tau in the hippocampus (FangFang et al., 2017). In young subjects, Nrf2 can counteract the vascular oxidative damage and augmented ROS production associated with obesity (Ungvari et al., 2011a; Csiszar et al., 2015). However, aging is associated with Nrf2/ARE signaling pathway dysfunction, increasing the susceptibility of the elderly to obesity-driven oxidative stress. As oxidative stress is already exacerbated in these individuals by various other factors, the dysfunctioning of Nrf2 signaling will further aggravate this pro-oxidative scenario and contribute to the development of neuropsychiatric disorders in the aging brain (Morrison et al., 2010; Ungvari et al., 2011b,c).

Morrison et al. (2010) showed that 20-month old male mice fed a HFD composed of 60% fat, but not 41% fat, presented increased hippocampal oxidative stress and cognitive decline after 4 months, when compared to controls. These changes were associated with a decrease in Nrf2 levels and activity, suggesting that Nrf2 signaling impairment may be a mechanism of HFD-induced cognitive dysfunction in the aging brain.

Interestingly, numerous studies have demonstrated that DER can counteract the negative effects of HFD and promote a wide array of beneficial effects on the health of overweight individuals (Mattson, 2012). For instance, obese or overweight individuals show improved cognitive function and mood regulation following 12 months of DER (low carbohydrate or fat intake) (Brinkworth et al., 2009).

A HFD can result in neuronal insulin resistance, a marker of diabetes, contributing to cognitive impairment. Moreover, AD risk is positively correlated to brain insulin resistance and diabetes (Pipatpiboon et al., 2012; Tucsek et al., 2014; FangFang et al., 2017). Studies in an AD murine model show that, unlike DER, HFD and T2D symptoms can aggravate Aβ deposition in the brain and contribute to cognitive impairment (Ho et al., 2004; Takeda et al., 2010). In an AD post-mortem study, insulin resistance positively correlated with Aβ plaques and negatively correlated with last recorded cognitive function (Talbot et al., 2012).

A recent study in triple transgenic AD mice showed that HFD (60% fat) for 4 months resulted in cognitive impairment, coupled to increased oxidative stress and neuronal cell death. These changes were attributed to suppressed Nrf2/ARE pathway activation along with reduced expression of the Nrf2 target genes HO-1 and manganese SOD (MnSOD) (Sah et al., 2017). Tarantini et al. (2018) fed Nrf2 knockout mice a HFD (60% fat) for 5 months, with Nrf2 deficiency significantly increasing HFD-induced brain inflammation, oxidative stress, synaptic disruption and BBB permeability, as well as raising levels of amyloid precursor protein (APP) gene expression, the proteolysis of which produces Aβ. The authors concluded that Nrf2 dysfunction exacerbates the obesity-induced adverse effects in the brain and plays a role in vascular cognitive impairment and AD (Tarantini et al., 2018).

As with AD, calorie intake throughout life can influence the incidence of PD in some individuals (Mattson, 2012). Several lines of evidence have shown that diet, adiposity and T2D are linked to PD (Logroscino et al., 1996; Anderson et al., 1999; Johnson et al., 1999; Abbott et al., 2002; Hu et al., 2006, 2007; Morris et al., 2011; Schernhammer et al., 2011; Xu et al., 2011). All these factors are correlated to a HFD, given that over 80% of T2D patients are overweight or obese, whilst a HFD is frequently used as a model of diabetes (Centers for Disease Control and Prevention (CDC), 2004; Morris et al., 2011).

Many studies have shown that a HFD renders SN neurons more vulnerable to PD neurotoxins and increased ROS levels (Choi et al., 2005; Morris et al., 2010, 2011; Bousquet et al., 2012). In one of these works, mice fed a HFD or control diet for 5 weeks were treated with the PD-related neurotoxin 6-OHDA, with the HFD mice presenting with higher oxidative stress and neurodegeneration (dopamine depletion in the striatum and SN). These poorer outcomes were all correlated with heightened levels of adiposity and insulin resistance (Morris et al., 2010).

Innate and adaptive immune systems have a pivotal role in MS pathogenesis. Nrf2 agonists are promising candidates in the treatment of MS, since CDDO-Im 1-[2-cyano-3-,12-dioxooleana-1,9(11)-dien-28-oyl] imidazole, a Nrf2 activator, promoting the differentiation of the less inflammatory Th2 t cell phenotype in stimulated splenocytes of C57BL/6 mice, thereby lowering INFγ and TNFα production as well as NF-κB DNA binding (Zagorski et al., 2018). However, it has more recently been proposed that inflammatory processes are secondary to primary cytodegenerative processes in some neural cells, such as oligodendrocytes and neurons, with some studies strongly suggesting that alterations in mitochondrial functioning, and consequent increase ROS, can drive the initial neurodegeneration in MS (Witte et al., 2014). Furthermore, increased oxidative stress is correlated with decreased Complex IV electron transport chain gene expression and Nrf2 activity in the non-lesioned gray matter of the frontal and parietal cortex, from post-mortem MS and control groups, suggesting that mitochondrial function is correlated with, if not regulated by, Nrf2 in MS (Pandit et al., 2009).

Epidemiological studies also indicate a positive correlation between MS severity and fatty acids intake, with a long-term, higher level saturated fat consumption being associated with an increased frequency of MS as well as augmented EAE symptomatology (Schwarz and Leweling, 2005; Thompson, 2008; Winer et al., 2009). Moreover, overweight and obese 20-year olds have a higher risk of developing MS compared to those of normal weight (Hedstrom et al., 2014). Likewise, several other dietary compounds like tea, coffee, alcohol and sweets are connected with MS incidence (Antonovsky et al., 1965; Berr et al., 1989; Sepcic et al., 1993; Tola et al., 1994). Timmermans and colleagues showed an increased clinical score and proinflammatory genes (IL6, INFγ, and IL1β), as well as higher levels of infiltrating CD3+ T cells in the CNS of female animals subjected to a HFD. Similarly, Fernandez-Real and Pickup (Fernandez-Real and Pickup, 2008) showed an increase in inflammatory cytokines, soluble adhesion molecules, and chemokines in the blood of obese people. Accordingly, the Nrf2 activator, CDDO-IM, prevented the body weight gain in animals submitted to HFD, partly by regulating expression of fatty acid synthesis and oxidation enzymes in the liver (Shin et al., 2009).

As in MS, a HFD also is a risk factor for cardiovascular diseases and cerebral ischemia. Recent research has revealed an increase in the prevalence of acute ischemic stroke in children and young adults, correlated with such risk factors, as obesity and lipid disorders (George et al., 2011). Impairment in vascular function is evident in male Wistar rats submitted to 8-week HFD before transient middle cerebral artery occlusion (MCAO), with HFD significantly increasing not only body weight and adiposity as well as associated processes, but also augmenting the infarct size in rats (Li et al., 2013). Such data highlights how alterations in the regulation of energy intake are intimately linked to levels of Nrf2, in the regulation of susceptibility to, and severity of, a wide array of seemingly distinct medical conditions.

Interestingly, both HFD and lack of Nrf2 can increase infarct area after cerebral ischemia. Deutsch et al. (2009) demonstrated that HFD-rats submitted to cerebral ischemia by MCAO showed smaller lumens and thicker MCA walls, when compared to normal diet controls. This is attributed to increased expression of metalloproteinase-2 expression and collagen-1 deposition, suggesting impairment on neurovascular functions (Deutsch et al., 2009; Osmond et al., 2010; Li et al., 2013). In another study, the volume infarct of Nrf2-deficient mice subjected to MCAO for 90 min and 24 h of reperfusion was increased when compared to the control group (Shah et al., 2007). Intracerebral ventricular pre-administration of tert-butylhydroquinone reduced the infarct area in the MCAO mouse brain (Shih et al., 2005). Corroborating these findings, pharmacological agonism of Nrf2, by dimethyl fumarate, reduced NF-κB activation and protected the calcium-activated potassium (BK) channel-mediated coronary vasodilatation in HFD mice (Lu et al., 2017). These findings suggest that both Nrf2 and HFD have important roles in neurovascular and cerebral ischemia modulation.

It is also of note that dietary impacts on the composition of the gut microbiome may be of some relevance to the data presented above. There is considerable interest in the role of gut microbiome changes in the etiology, course and treatment of AD, PD, and MS (Anderson and Maes, 2017), mediated via changes in the gut-brain and gut-liver axes. By increasing gut permeability, gut bacteria and tiny fragments of partially digested food can trigger an immune reaction, with a wide array of consequences, including the possible production of α-synuclein in the gut, and its transport via neurons to the brain in the etiology of PD (Chen et al., 2018). Generally, an increase in the gut bacteria producing the small chain fatty acid, butyrate, is beneficial across a wide array of medical conditions. It is of note that butyrate increases levels of Nrf2 (Anderson et al., 2016), suggesting that some of the benefits of butyrate may be mediated not only by its histone deacetylase inhibitor capacity and its induction of melatonin, but also by its induction of Nrf2 (Dong W. et al., 2017).

## Conclusion

It is widely accepted that oxidative stress plays a central role in neurological disorders. This underpins the importance of targeting Nrf2 to counteract such oxidative stress and associated brain diseases (Patel, 2016). Dietary interventions such as DER protocols, in contrast to a HFD, can promote small energetic challenges to the brain that enhance Nrf2/ARE pathway activation (Mattson, 2012). Nrf2 up-regulates the expression of several pro-survival genes and counteracts oxidative damage to the CNS, thereby preventing neurodegeneration and obesity-related brain disorders (Calkins et al., 2009). Hence, DER may be a valuable treatment option for brain disorders, including as adjunct therapy with other known Nrf2 activators. However, controversial reports showed that NRF2 activation can result in drug resistance and oncogenic effects (Sporn and Liby, 2012). Thus, additional large-scale studies are warranted to further explore the effects of dietary interventions in Nrf2/ARE signaling and to establish the best dietary protocols for humans to optimize the beneficial effects of Nrf2 for the prevention and/or early intervention in the etiology and course of neurological disorders.

## Author Contributions

AV, NdS, CS, and CM contributed to the design of the manuscript, literature review, writing of the manuscript, and creation of the figures.

## Conflict of Interest Statement

The authors declare that the research was conducted in the absence of any commercial or financial relationships that could be construed as a potential conflict of interest.

## Abbreviations

6-OHDA: 6-hydroxydopamine
Aβ: Amyloid β
AD: Alzheimer’s disease
ARE: antioxidant response element
BBB: blood-brain barrier
BMI: body mass index
CAT: catalase
CNS: central nervous system
CR: calorie restriction
DAMPS: damage-associated molecular patterns
DCs: dendritic cells
DER: dietary energy restriction
EAE: experimental autoimmune encephalomyelitis
GPx: glutathione peroxidase
GR: glutathione reductase
GST: glutathione S-transferase
HFD: high-fat diet
HO-1: heme oxygenase 1
IF: intermittent fasting
KEAP1: Kelch-like ECH associated protein 1
MCA: middle cerebral artery
MCAO: middle cerebral artery occlusion
MnSOD: manganese superoxide dismutase
MPTP: methyl-4-phenyl-1, 2, 5, 6-tetrahydropyridine
MS: multiple sclerosis
NQO1: NADPH quinine oxidoreductase 1
Nrf2: Nuclear factor erythroid 2-related factor 2
OGDR: oxygen-glucose deprivation/reoxygenation
OPCs: oligodendrocytes and oligodendrocytes precursor cells
PD: Parkinson’s disease
ROS: reactive oxygen species
RRMS: relapsing-remitting multiple sclerosis
SN: substantia nigra
SOD: superoxide dismutase
SPMS: secondary progressive multiple sclerosis
T2D: type 2 diabetes
tMCAO: transient middle cerebral artery occlusion
VEGF: vascular endothelial growth factor
